# Downregulation of lncRNA MNX1-AS1 promotes the ferroptosis and apoptosis of non-small cell lung cancer

**DOI:** 10.7150/ijms.97790

**Published:** 2025-02-03

**Authors:** Xinming Chi, Lingya Feng, Longzhu Wang, Shijie Yu, Minna Wei, Qianran Zhang, Xuefeng Liu, Shujuan Shao

**Affiliations:** 1 University Key Laboratory of Proteomics in Liaoning Province, Dalian Medical University, Dalian, China.; 2 Department of Pathology, Second Affiliated Hospital of Dalian Medical University, Dalian, China.; 3 Institute of Cancer Stem Cell, Dalian Medical University, Dalian, China.

## Abstract

Non-small cell lung cancer (NSCLC), the main histological type of lung cancer, poses a serious threat to human health. Increasing evidence has shown that long non-coding RNA (lncRNA) MNX1-AS1 is involved in the development and progression of cancers, including lung cancer. Apoptosis and ferroptosis, which are two forms of regulated cell death, can be induced by anti-cancer drugs. However, the roles of MNX1-AS1 in apoptosis and ferroptosis are still unclear. Here, we found that knockdown of MNX1-AS1 promoted the ferroptosis induced by RSL3 in NSCLC cells, with a decrease in cell viability and increases in reactive oxygen species (ROS) and malondialdehyde (MDA) levels. Meanwhile, acridine orange/ethidium bromide (AO/EB) double staining, terminal deoxynucleotidyl transferase-mediated dUTP nick-end labeling (TUNEL) assay and Annexin V/PI double staining revealed that knockdown of MNX1-AS1 promoted the apoptosis caused by paclitaxel in NSCLC cells. In addition, knockdown of MNX1-AS1 resulted in increased expression of pro-apoptotic protein BAX as well as the cleaved caspase-3 and PARP1, and decreased expression of anti-apoptotic protein Bcl-2. RNA sequencing and quantitative real-time PCR assay identified that the expression of ACSL4 was increased, while the expression of ABCG2 was reduced when MNX1-AS1 was knocked down. Rescue assay showed that ACSL4 and ABCG2 were involved in MNX1-AS1-mediated ferroptosis and apoptosis, respectively. Furthermore, knockdown of MNX1-AS1 increased the sensitivity of NSCLC cells to the combination of RSL3 and paclitaxel. Taken together, our data suggest that MNX1-AS1 might be a potential therapeutic target for lung cancer, especially in combination of ferroptosis and/or apoptosis-inducing drugs.

## Introduction

Lung cancer remains one of the deadliest human cancers worldwide, which ranks second in incidence and first in mortality, accounting for approximately 11.4% of new cancer cases and 18.0% of cancer deaths in 2020 1. Non-small cell lung cancer (NSCLC) is the primary histological subtype of lung cancer, but the treatment options for NSCLC are still limited 2. Thus, it is urgent to explore new therapeutic targets and strategies for lung cancer.

Apoptosis and ferroptosis represent two forms of regulated cell death (RCD) 3. Ferroptosis is a novel type of RCD driven by iron-dependent lipid peroxidation, which is different from cell apoptosis 4-7. Although apoptosis and ferroptosis are morphologically and genetically distinct from each other, they are not mutually exclusive. Some genes and agents have the ability to induce both apoptosis and ferroptosis. For example, pathways such as FBW7-NRA41-SCD1 axis have be reported to play a synchronous role in apoptosis and ferroptosis in pancreatic cancer cells 8. Moreover, it is recently reported that one of the natural ent-Kaurane diterpenoids could induce apoptosis and ferroptosis by destructing the intracellular redox homeostasis 9. These studies suggest that synergistic induction of apoptosis and ferroptosis may be an effective strategy for killing cancer cells.

Long non-coding RNA (lncRNA) is a class of RNA that is more than 200 nucleotides in length and does not encode a protein. Increasing evidence has shown that lncRNA plays essential roles in gene expression through multiple ways, such as recruiting transcription factors to regulate transcription 10, mediating stress granule formation to modulating translation 11, and acting as a molecular sponge to regulate mRNA abundance 12. Notably, as an important functional molecule, lncRNA is involved in cancer development and progression 10,12-14. LncRNA MNX1-AS1 has been reported to be upregulated in a variety of human cancers, contribute to cancer cell proliferation, migration and invasion, and inhibit cancer cell apoptosis 15-19. High MNX1-AS1 expression is associated with poor overall survival of cancer patients 15-19. MNX1-AS1 is also highly expressed in lung cancer and depletion of MNX1-AS1 inhibits lung cancer cell growth, migration and invasion, and promotes cell apoptosis 20-22. Interestingly, a recent study has showed that MNX1-AS1 interacts with IGF2BP1 and promotes its phase separation, thus enhancing the stability of c-Myc and E2F1 mRNA and the proliferation of lung cancer cells 23. However, the effect of MNX1-AS1 on chemotherapy drug-induced apoptosis, especially on ferroptosis in lung cancer, is not yet clear.

In this study, we found that MNX1-AS1 could regulate ferroptosis and apoptosis in NSCLC cells. Knockdown of MNX1-AS1 promoted RSL3-induced ferroptosis and paclitaxel-induced apoptosis. Through RNA sequencing, bioinformatics analyses and rescue assays, we identified ACSL4 and ABCG2 as downstream genes of MNX1-AS1, which were required for MNX1-AS1 depletion-caused ferroptosis and apoptosis, respectively. Our data suggest that MNX1-AS1 might serve as a potential target for increasing sensitivity to chemotherapy drugs.

## Materials and Methods

### Cell culture

Human lung cancer cell lines A549, NCI-H460 and NCI-H1299 were obtained from American Type Culture Collection (ATCC, USA), while NCI-H1975, HCC827, NCI-H446 and NCI-H2170 were obtained from National Collection of Authenticated Cell Cultures (Shanghai, China). All cells were cultured in RPMI-1640 (Gibico) medium containing 10% fetal bovine serum (FBS) in a humidified incubator at 37℃ with 5% CO_2_. All cell lines were authenticated by short tandem repeat (STR) profiling before used.

### Stable knockdown cell line construction

Lentiviruses expressing MNX1-AS1-specific short hairpin RNA (shRNA) and control lentiviruses were obtained from Shanghai Genechem Co., Ltd. (China). A549 and H1975 cells were infected with these lentiviruses in the presence of HiTransG P. Puromycin (5 μg/mL) was then used to select the stable knockdown cell lines and their corresponding control cells. The shRNA sequences targeting MNX1-AS1 are as follows: shRNA-1, 5′-ACACAGGGAAGAAGCACAAAT-3′; shRNA-2, 5′-AGCCACCAAACACATGCATAA-3′.

### RNA interference and plasmid transfection

Small interfering RNA (siRNA) targeting ACSL4 was obtained from Shanghai GenePharma Co., Ltd. (China). The sequences of ACSL4 siRNA are as follows: sense 5′-GACCGAAGGACACAUAUAUTT-3′, antisense 5′-AUAUAUGUGUCCUUCGGUCTT-3′. Flag-tagged ABCG2 plasmid was purchased from Shanghai Genechem Co., Ltd. (China). A549 control cells and MNX1-AS1-knockdown cells were transiently transfected with either siRNA by using Lipofectamine 2000 reagent (Invitrogen, USA) or plasmid by using Neofect™ DNA transfection reagent (Neofect (beijing) biotech Co., Ltd., China), according to the kit's instructions.

### Cell proliferation and drug sensitivity assays

For cell proliferation assay, 1500 cells were seeded into each well of 96-well plates and cultured for 96 hours. 10 μL of CCK-8 solution (Apexbio, USA) was added to each well every 24 hours. After incubation at 37℃ for 1 hour, the absorbance value was detected at 450 nm.

For drug sensitivity assay, 3000 cells were seeded into each well of 96-well plates. After cultured for 24 hours, the cells were treated with increasing doses of RSL3 or paclitaxel for 24 hours. MTT solution (0.5 mg/mL; Coolaber, China) was added to each well and incubated at 37℃ for 4 hours. After removing the medium, DMSO was added and the absorbance value was detected at 492 nm.

### Colony formation assay

Cells were trypsin-digested into single cell suspension and seeded into six-well plates. After about 14 days, cell colonies were fixed with 4% paraformaldehyde, stained with crystal violet solution, and photographed.

### Animal models

Female BALB/c nude mice (4-6 weeks old) were purchased from Liaoning Changsheng biotechnology co., Ltd. (China). 2 × 10^6^ of A549 control cells or MNX1-AS1-knockdown cells were inoculated subcutaneously into the right axilla of the mice (n = 6/group). The mice were then routinely fed for 18 days. The tumor volume was monitored with a caliper and calculated as follows: volume = 0.5 × length × width^2^. At the end of the experiment, the mice were sacrificed and the xenograft tumors were excised, measured, and photographed. Moreover, the xenograft tumors were fixed, paraffin-embedded, and immunohistochemically stained with anti-Ki67 (1:100; Proteintech), anti-4-HNE (1:100; Bioss), anti-BAX (1:100; Proteintech) or anti-cleaved PARP1 (1:100; Abcam) antibody. The Animal Care and Use Committee of Dalian Medical University approved the animal experiments.

### Measurement of intracellular reactive oxygen species (ROS)

Cells with a density of 60%-70% were treated with RSL3 for 24 hours. Serum-free medium containing DCFH-DA probe (10 μM; Beyotime Biotechnology, China) was added to the cells and incubated for 30 minutes at 37°C. The cells were washed three times with PBS and, finally, were visualized under a fluorescence microscope (Olympus, Japan).

### Measurement of malondialdehyde (MDA) level

Cells with a density of 60%-70% were treated with RSL3 for 24 hours. Then, the level of MDA was detected by Cell Malondialdehyde (MDA) Assay Kit (Colorimetric method) (Nanjing Jiancheng Bioengineering Institute, China) according to the kit's instructions. The absorbance was measured at 532 nm.

### Acridine orange/ethidium bromide (AO/EB) double staining

Apoptotic cells were detected by using AO/EB double staining method with Normal/Apoptotic/Necrotic Cell Detection Kit (KeyGEN BioTECH, China) according to the manufacturer's instructions. Cells with a density of 60%-70% were treated with paclitaxel for 24 hours. After washed two times with PBS, the cells were trypsin-digested into single cell suspension, incubated with a mixture of AO and EB solutions, and finally observed under a fluorescence microscopy (Olympus, Japan).

### Terminal deoxynucleotidyl transferase-mediated dUTP nick-end labeling (TUNEL) assay

Apoptotic cells were also detected by using One Step TUNEL Apoptosis Assay Kit (Beyotime Biotechnology, China) according to the manufacturer's instructions. Cells with a density of 60%-70% were treated with paclitaxel for 24 hours. After washed with PBS, the cells were fixed with 4% paraformaldehyde for 30 minutes, permeabilzed by 0.3% Triton X-100 in PBS for 5 minutes, and incubated with TUNEL detection solution for 60 minutes at 37°C. Then, the cells were stained with DAPI at room temperature for 10 minutes and were observed under a fluorescence microscope (Olympus, Japan).

### Annexin V/PI double staining

Apoptotic cells were also evaluated by using Annexin V-FITC/PI Apoptosis Detection Kit (KeyGEN BioTECH, China) according to the manufacturer's instructions. Cells with a density of 60%-70% were treated with paclitaxel for 24 hours. After washed two times with PBS, the cells were trypsin (without EDTA)-digested into single cell suspension. Then, cells were washed twice with PBS and incubated with Annexin V-FITC and PI. After 5-10 minutes, the fluorescence intensity was detected by flow cytometry.

### RNA sequencing and analysis

Total RNAs of A549 control cells and MNX1-AS1-knockdown cells were isolated with RNAiso Plus (Takara). RNA sequencing and data analysis were performed by Novogene Bioinformatics Technology Co., Ltd. (China). Briefly, after mRNA was purified using poly-T oligo-attached magnetic beads, the library was constructed and sequenced on Illumina Novaseq platform to generate 150 bp paired-end reads. Clean data were obtained by removing low quality reads and reads containing adapter or N base. Fragments per kilobase of transcript per million mapped reads (FPKM) were calculated and differential expression analysis was performed using the DESeq2 R package (1.20.0). *P* value was adjusted by using Benjamini & Hochberg method. Genes with adjusted *P* < 0.05 were considered as differentially expressed genes (DEGs). Gene Ontology (GO) enrichment analysis of DEGs was conducted on the DAVID website 24,25.

### Reverse transcription-quantitative real-time polymerase chain reaction (RT-qPCR)

Total RNA was extracted from cells by using RNAiso Plus (Takara) and was subsequently reverse-transcribed into first-strand cDNA with a reverse transcription kit (abm, Canada) according to the manufacturer's manual. Quantitative real-time PCR (qPCR) was performed by using MonAmp™ ChemoHS qPCR Mix (Monad, China). The relative expression levels of target genes were calculated with the 2^-ΔΔCt^ methods. GAPDH was used as an endogenous control. The sequences of primers used are listed in Supplemental Table S1.

### Western blot

Cells were lysed by using RIPA Lysis Buffer (Beyotime Biotechnology, China) containing protease inhibitors, and the supernatant was collected by centrifugation at 12,000 rpm for 20 minutes at 4 °C. The concentration of protein samples was determined by using BCA Protein Assay Kit (Beyotime Biotechnology, China). Protein samples (30 μg/lane) were separated on sodium dodecyl sulfate-polyacrylamide gel electrophoresis (SDS-PAGE) and were then transferred to nitrocellulose membrane (Millipore, USA). After blocked with 5% skimmed milk for 1 hour at room temperature, the membrane was incubated with primary antibody against caspase-3 (1:1000; Proteintech), cleaved caspase-3 (1:1000; Abcam), BAX (1:1000; Proteintech), PARP1 (1:1000; Proteintech), Bcl-2 (1:1000, Proteintech), ACSL4 (1:1000, Abcam), ABCG2 (1:1000; Abcam), β-actin (1:5000, Proteintech) or GAPDH (1:5000, Proteintech) at 4 °C overnight. Next day, the membranes were further incubated with horseradish peroxidase-labeled anti-mouse or anti-rabbit IgG secondary antibody at room temperature for 1 hour. Protein bands were visualized by using enhanced chemiluminescence reagents (New Cell & Molecular Biotech Co., Ltd., China) on ChemiDoc™ XRS+ system (Bio-Rad, USA).

### Statistical analysis

Data were statistically analyzed by using GraphPad Prism 8.0 software. All quantitative results were expressed as mean ± S.D. Fluorescence images were analyzed by using Image J software. Statistical significance was performed using two-tailed Student's *t*-test. *P* < 0.05 was considered statistically significant.

## Results

### Knockdown of MNX1-AS1 inhibits the proliferation of NSCLC cells

**Figure 1:**
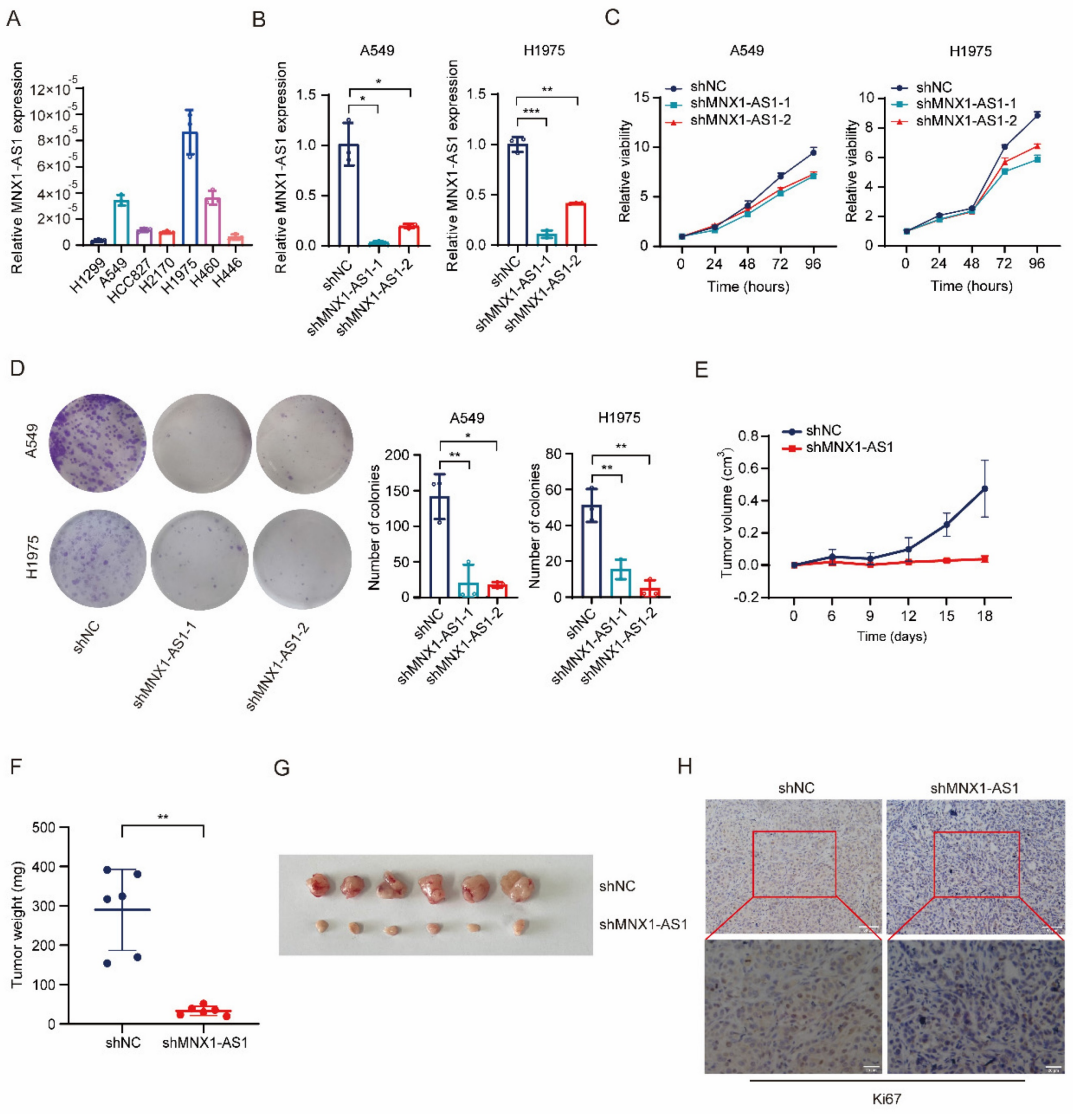
** Knockdown of MNX1-AS1 inhibits the proliferation of NSCLC cells.** (**A**) RT-qPCR assay detecting the expression level of MNX1-AS1 in seven lung cancer cell lines. (**B**) The stable knockdown efficiency of MNX1-AS1 in A549 and H1975 cell lines was assessed by RT-qPCR. (**C**, **D**) The viability and colony formation ability of A549 (or H1975) control cells and MNX1-AS1-knockdown cells were detected by CCK-8 assay (**C**) and colony formation assay (**D**), respectively. (**E**-**G**) A549 control cells and MNX1-AS1-knockdown cells were inoculated subcutaneously into nude mice. The volume and weight of xenograft tumors were measured. (**H**) Immunohistochemical staining for Ki67 was performed in xenograft tumors formed by A549 control cells and MNX1-AS1-knockdown cells. Data were presented as mean ± S.D. *P < 0.05, **P < 0.01, ***P < 0.001.

To investigate the role of MNX1-AS1 in NSCLC proliferation, we detected the expression of MNX1-AS1 in several lung cancer cell lines, and then selected A549 and H1975, which exhibited high MNX1-AS1 expression, to stably knock down MNX1-AS1 via lentivirus-mediated delivery of shRNA (Fig. 1A and B). CCK-8 and colony formation assays on these MNX1-AS1-knockdown cells showed that knockdown of MNX1-AS1 significantly inhibited the viability and colony formation ability of both A549 and H1975 cells (Fig. 1C and D). To confirm the contribution of MNX1-AS1 to NSCLC proliferation *in vivo*, we subcutaneously inoculated nude mice with A549 control cells and MNX1-AS1-knockdown cells, respectively. As shown in Fig. 1E-G, the xenograft tumors formed by MNX1-AS1-knockdown cells were much smaller than those of control group, indicating that knockdown of MNX1-AS1 significantly reduced the tumorigenic ability of A549 cells *in vivo*. Immunohistochemical staining of tumor tissues showed that Ki67 level was reduced after the knockdown of MNX1-AS1 (Fig. 1H). In summary, these findings demonstrated that the knockdown of MNX1-AS1 inhibits the proliferation of NSCLC cells *in vitro* and *in vivo*.

### Knockdown of MNX1-AS1 increases ferroptosis in NSCLC cells

**Figure 2:**
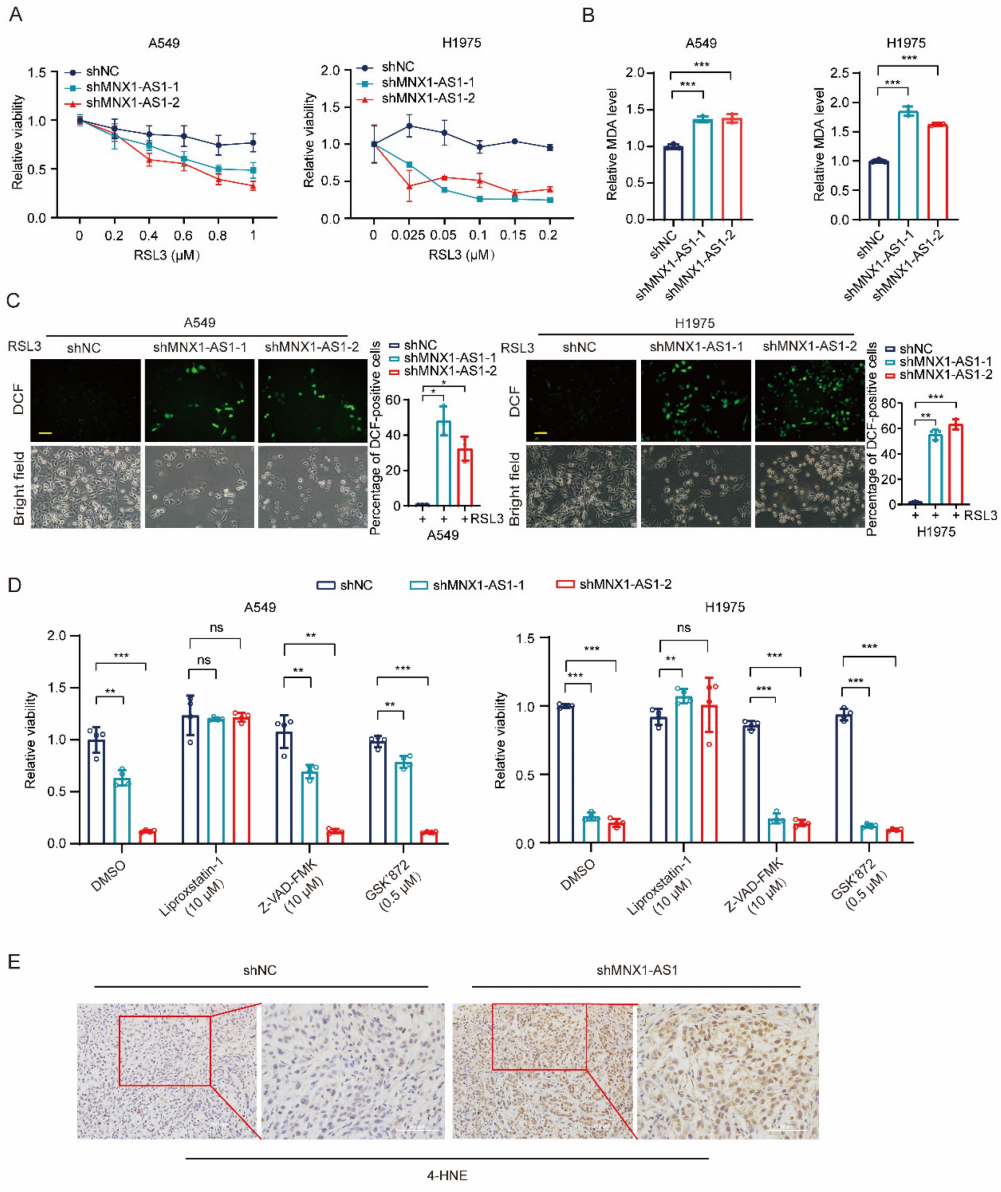
**Knockdown of MNX1-AS1 increases ferroptosis in NSCLC cells.** (**A**) A549 (or H1975) control cells and MNX1-AS1-knockdown cells were treated with different concentrations of RSL3. After 24 hours of treatment, the cell viability was detected by MTT assay. (**B**, **C**) The MDA and ROS levels in A549 (or H1975) control cells and MNX1-AS1-knockdown cells treated with RSL3 (A549: 0.6 μM; H1975: 0.2 μM) for 24 hours were detected using MDA assay kit (**B**) and DCFH-DA probe (**C**), respectively. Scale bar: 50 μm. (**D**) A549 (or H1975) control cells and MNX1-AS1-knockdown cells were treated with RSL3 together with the ferroptosis inhibitor Liproxstatin-1, apoptosis inhibitor Z-VAD-FMK or necrosis inhibitor GSK'872. After treatment for 24 hours, the cell viability was detected by MTT assay. (**E**). Immunohistochemical staining for 4-HNE was performed in xenograft tumors formed by A549 control cells and MNX1-AS1-knockdown cells. Data were presented as mean ± S.D. *P < 0.05, **P < 0.01, ***P < 0.001; ns: non-significant.

To investigate the role of MNX1-AS1 in NSCLC ferroptosis, we induced ferroptosis by using different concentrations of RSL3 in A549 (or H1975) control cells and MNX1-AS1-knockdown cells. After 24 hours, MTT assay was performed and showed that the viability of MNX1-AS1-knockdown A549 (or H1975) cells was lower than that of control cells upon RSL3 induction, indicating that knockdown of MNX1-AS1 promotes RSL3-induced cell death (Fig. 2A). To evaluate the effect of MNX1-AS1 knockdown on lipid peroxidation, we detected the levels of intracellular MDA in A549 (or H1975) control cells and MNX1-AS1-knockdown cells upon RSL3 induction. The results showed that knockdown of MNX1-AS1 led to an increase in MDA levels in both A549 and H1975 cells (Fig. 2B). Subsequently, we measured intracellular ROS levels using DCFH-DA probe and found that knockdown of MNX1-AS1 also enhanced ROS production in A549 and H1975 cells under RSL3 treatment (Fig. 2C). To further confirm the role of MNX1-AS1 in ferroptosis, we used the ferroptosis inhibitor Liproxstatin-1, the apoptosis inhibitor Z-VAD-FMK or the necrosis inhibitor GSK'872 together with RSL3 to treat A549 (or H1975) control cells and MNX1-AS1-knockdown cells. As expected, Liproxstatin-1, instead of Z-VAD-FMK and GSK'872, could reverse the death of MNX1-AS1-knockdown cells under RSL3 treatment (Fig. 2D). Additionally, immunohistochemical staining of xenograft tumors showed that the level of 4-hydroxynonenal (4-HNE), a lipid peroxidation marker, was increased upon MNX1-AS1 knockdown (Fig. 2E). Collectively, these results suggest that the knockdown of MNX1-AS1 promotes the RSL3-induced ferroptosis in NSCLC cells.

### Knockdown of MNX1-AS1 increases apoptosis in NSCLC cells

**Figure 3:**
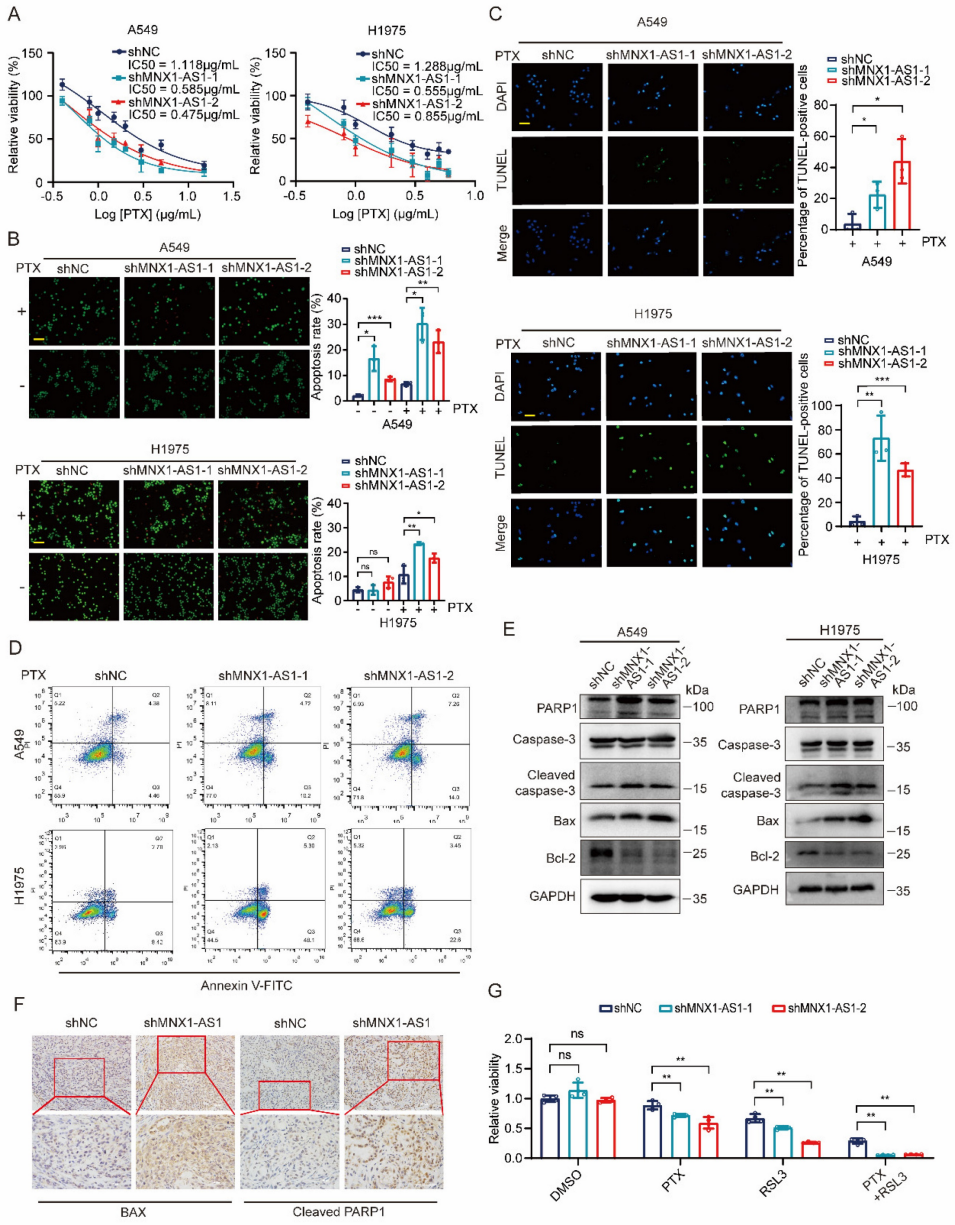
**Knockdown of MNX1-AS1 increases apoptosis in NSCLC cells.** (**A**) A549 (or H1975) control cells and MNX1-AS1-knockdown cells were treated with different concentrations of paclitaxel (PTX). After 24 hours of treatment, the cell viability was detected by MTT assay. (**B**) A549 (or H1975) control cells and MNX1-AS1-knockdown cells were treated with or without paclitaxel (1 μg/mL) for 24 hours. The apoptosis rate was detected by AO/EB double staining. Scale bar: 50 μm. (**C**, **D**) A549 (or H1975) control cells and MNX1-AS1-knockdown cells were treated with paclitaxel for 24 hours. The apoptosis rate was detected by TUNEL assay (**C**) and Annexin V/PI double staining (**D**). Scale bar: 50 μm. (**E**) Western blot detecting the expression of apoptosis-related proteins in A549 (or H1975) control cells and MNX1-AS1-knockdown cells. GAPDH was used as the loading control. (**F**) Immunohistochemical staining for BAX and cleaved PARP1 was performed in xenograft tumors formed by A549 control cells and MNX1-AS1-knockdown cells. (**G**) A549 control cells and MNX1-AS1-knockdown cells were treated with RSL3, paclitaxel, or a combination of RSL3 and paclitaxel. After treatment for 24 hours, the cell viability was detected by MTT assay. Data were presented as mean ± S.D. *P < 0.05, **P < 0.01, ***P < 0.001; ns: non-significant.

Next, we evaluated whether knockdown of MNX1-AS1 could promote the apoptosis of NSCLC cells. To test it, we used different concentrations of paclitaxel to treat A549 (or H1975) control cells and MNX1-AS1-knockdown cells for 24 hours. MTT assay showed that MNX1-AS1-knockdown A549 (or H1975) cells exhibited decreased IC50 for paclitaxel compared with control cells (Fig. 3A). Then, we performed AO/EB double staining to detect the apoptotic rate of A549 (or H1975) control cells and MNX1-AS1-knockdown cells with or without paclitaxel treatment. The results showed that knockdown of MNX1-AS1 promoted paclitaxel-induced apoptosis in A549 and H1975 cells (Fig. 3B). Similarly, TUNEL assay revealed that knockdown of MNX1-AS1 resulted in an increase in apoptosis induced by paclitaxel (Fig. 3C). Consistent results were also observed by Annexin V/PI double staining followed by flow cytometry (Fig. 3D). To further confirm the promotive effect of MNX1-AS1 knockdown on apoptosis, we examined the expression of apoptosis-related proteins in A549 (or H1975) control cells and MNX1-AS1-knockdown cells. The expression of pro-apoptotic protein BAX as well as the cleaved caspase-3 and PARP1 was up-regulated, while the expression of anti-apoptotic protein Bcl-2 was down-regulated in MNX1-AS1-knockdown cells, as compared with the control cells (Fig. 3E).

Consistently, BAX and cleaved PARP1 levels were increased in xenograft tumors formed by MNX1-AS1-knockdown cells (Fig. 3F). These data collectively suggest that knockdown of MNX1-AS1 promotes paclitaxel-induced apoptosis in NSCLC cells. Based on the roles of MNX1-AS1 in both ferroptosis and apoptosis in NSCLC cells, we treated A549 control cells and MNX1-AS1-knockdown cells with RSL3 and paclitaxel together. Surprisingly, knockdown of MNX1-AS1 led to lower cell viability upon the combination of RSL3 and paclitaxel, compared to RSL3 or paclitaxel alone (Fig. 3G), indicating that MNX1-AS1 could participate in ferroptosis and apoptosis simultaneously.

### MNX1-AS1 regulates the expression of ACSL4 and ABCG2

**Figure 4:**
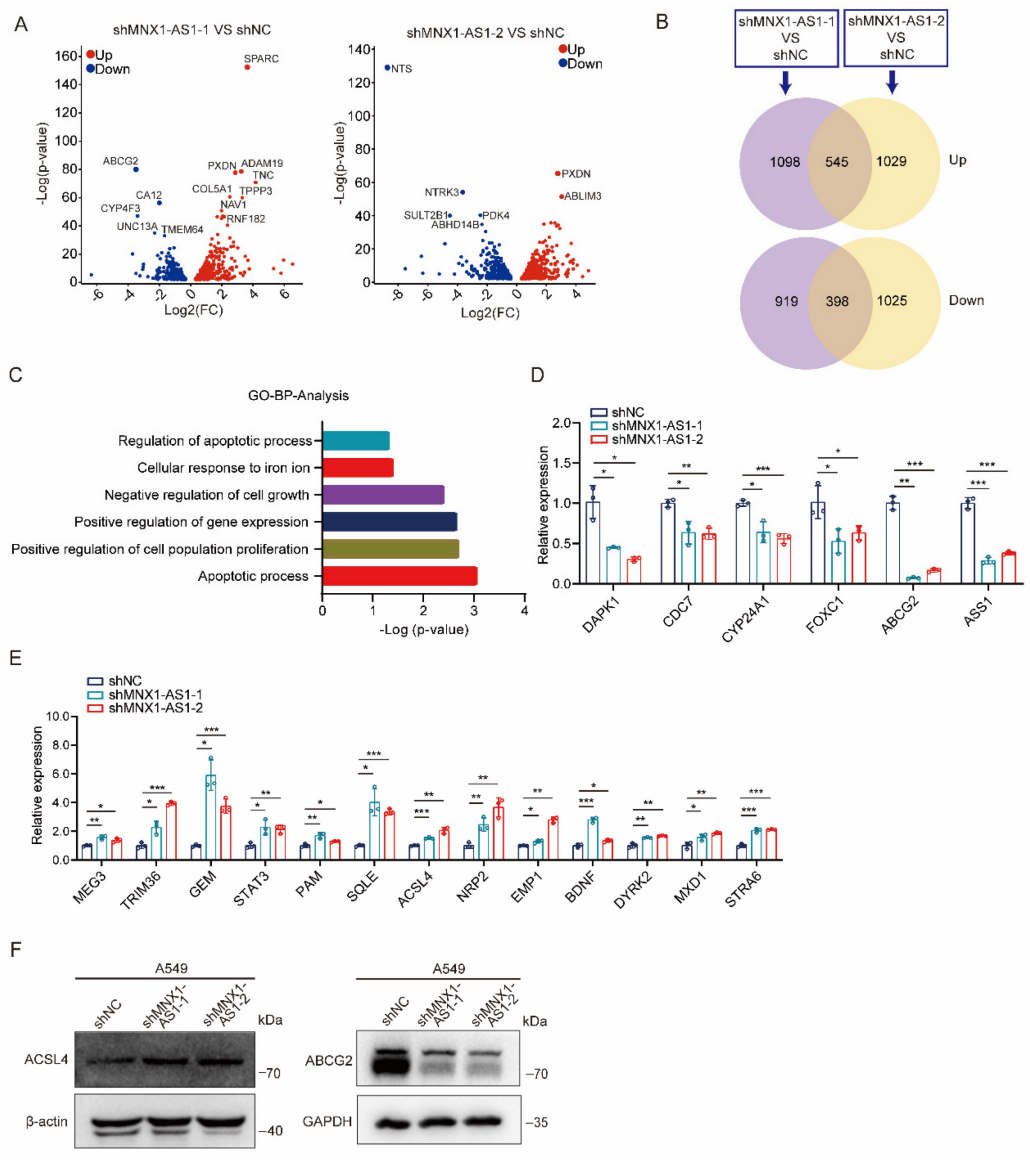
** MNX1-AS1 regulates the expression of ACSL4 and ABCG2.** (**A**) Volcano plots showing the DEGs overlapped in the two MNX1-AS1-knockdown A549 cell lines. The red and blue points indicate the up- and down-regulated DEGs, respectively. FC, Fold Change. (**B**) Venn diagrams showing the number of DEGs overlapped in the two MNX1-AS1-knockdown A549 cell lines. (**C**) GO enrichment analysis of the overlapped DEGs. Functions related to proliferation, ferroptosis or apoptosis are shown. (**D**, **E**) RT-qPCR validating the expression of down-regulated (**D**) and up-regulated (**E**) DEGs in A549 control cells and MNX1-AS1-knockdown cells. (**F**) Western blot detecting the expression of ACSL4 and ABCG2 in A549 control cells and MNX1-AS1-knockdown cells. β-actin or GAPDH was used as the loading control. Data were presented as mean ± S.D. *P < 0.05, **P < 0.01, ***P < 0.001.

To explore the molecular mechanisms by which MNX1-AS1 affects ferroptosis and apoptosis in NSCLC cells, we performed transcriptome sequencing on A549 control cells and MNX1-AS1-knockdown cells. By analyzing the sequencing data, a total of 943 DEGs were identified (with 545 up-regulated genes and 398 down-regulated genes) in both shMNX1-AS1-1 VS shNC group and shMNX1-AS1-2 VS shNC group (Fig. 4A and B). GO analysis showed that these DEGs could be enriched in functions related to proliferation, ferroptosis or apoptosis (Fig. 4C). We then used RT-qPCR to validate some significantly down-regulated or up-regulated genes that have been reported to participate in proliferation, ferroptosis or apoptosis (Fig. 4D and E). Among them, ACSL4 is an important ferroptosis-regulated gene 26-28, while ABCG2 is a multidrug efflux transporter that has been reported to be related to paclitaxel resistance 29. Therefore, we evaluated the protein levels of ACSL4 and ABCG2 and found that knockdown of MNX1-AS1 led to an increase in ACSL4 protein level and a decrease in ABCG2 protein level (Fig. 4F), which was consistent with their mRNA changes.

### Depletion of ACSL4 inhibits ferroptosis caused by MNX1-AS1 knockdown

**Figure 5:**
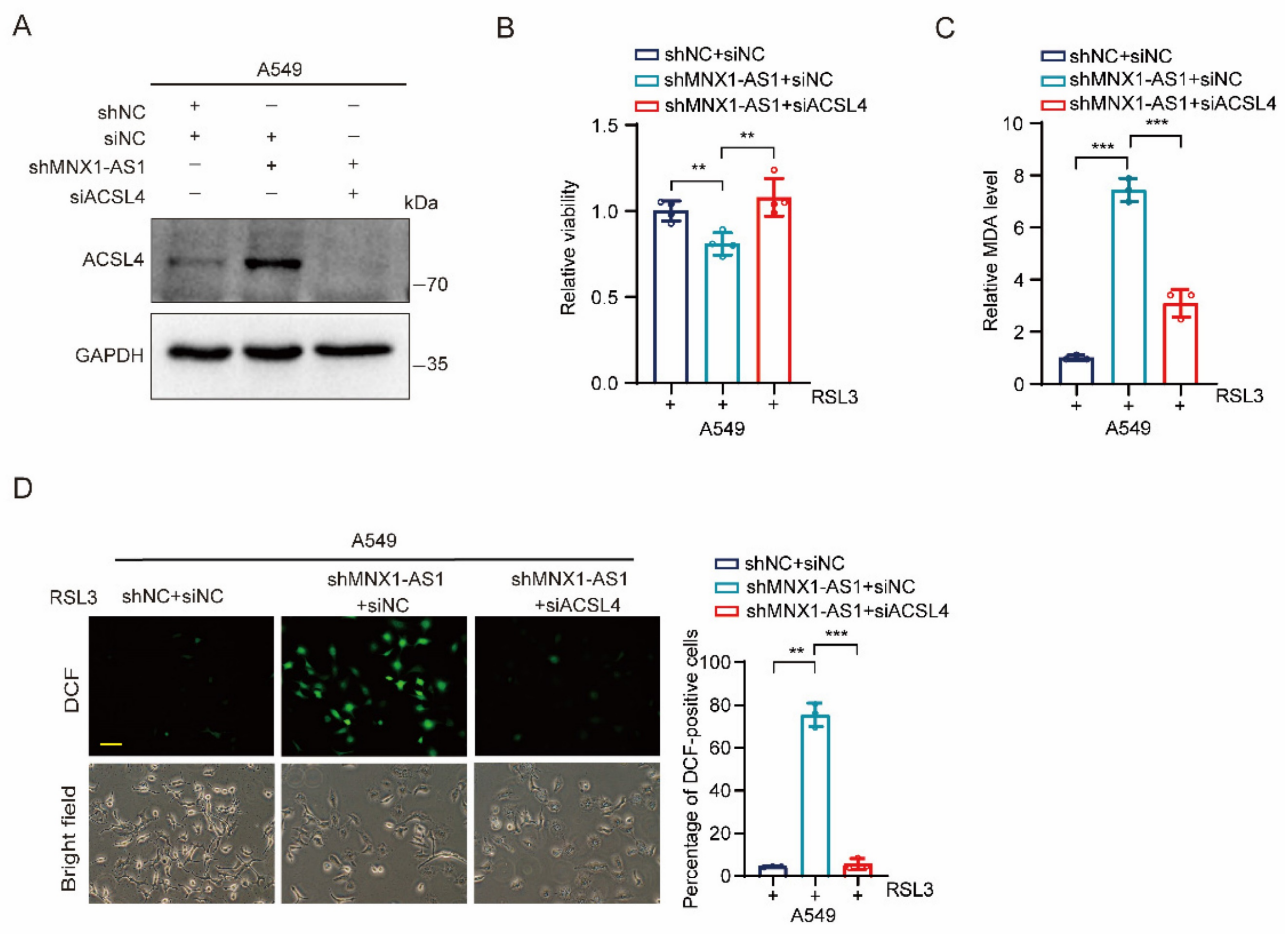
** Depletion of ACSL4 inhibits ferroptosis caused by MNX1-AS1 knockdown.** (**A**) ACSL4 siRNA or negative control siRNA was transiently transfected into A549 control cells or MNX1-AS1-knockdown cells. The knockdown efficiency of ACSL4 was then assessed by Western blot. GAPDH was used as the loading control. (**B**-**D**) A549 control cells and MNX1-AS1-knockdown cells transfected with ACSL4 siRNA or negative control siRNA were treated with RSL3 for 24 hours. The cell viability (**B**), MDA level (**C**) and ROS level (**D**) were detected by MTT assay, MDA assay kit and DCFH-DA probe, respectively. Scale bar: 50 μm. Data were presented as mean ± S.D. **P < 0.01, ***P < 0.001.

Because ACSL4 plays an essential role in ferroptosis, we hypothesized that ACSL4 may be involved in MNX1-AS1-regulated ferroptosis in NSCLC cells. To test it, we synthesized siRNA targeting ACSL4 and transfected it to MNX1-AS1-knockdown A549 cells to verify the silencing efficiency (Fig. 5A). Then, we treated these transfected cells with RSL3 for 24 hours followed by cell viability, MDA and ROS detection. MTT assay showed that depletion of ACSL4 restored the cell viability inhibited by MNX1-AS1 knockdown (Fig. 5B). Consistently, depletion of ACSL4 also suppressed the up-regulation of MDA and ROS caused by MNX1-AS1 knockdown (Fig. 5C and D). Taken together, these data suggest that ACSL4 is required for MNX1-AS1 knockdown-caused ferroptosis in NSCLC cells.

### Ectopic expression of ABCG2 inhibits apoptosis caused by MNX1-AS1 knockdown

**Figure 6:**
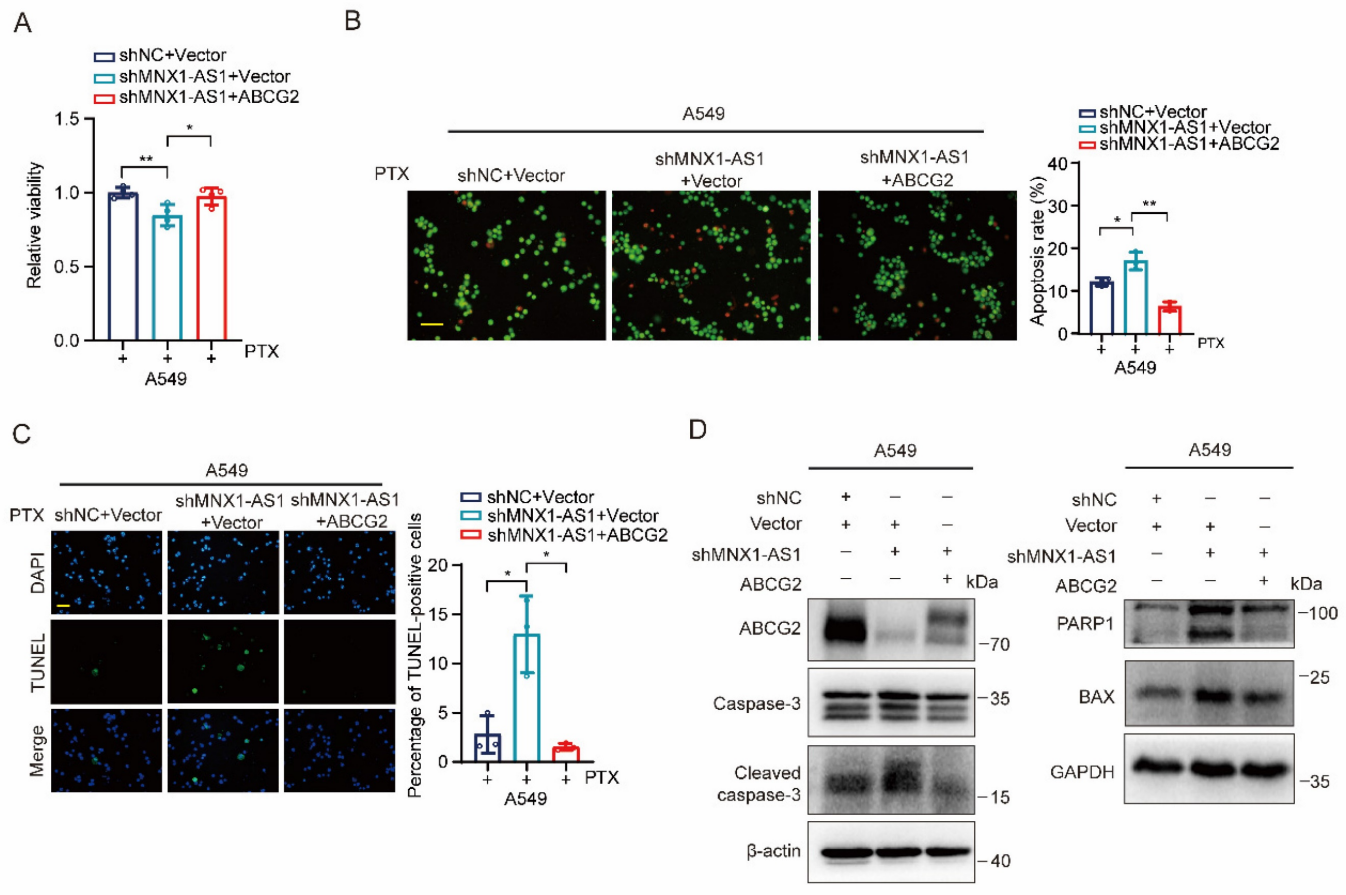
** Ectopic expression of ABCG2 inhibits apoptosis caused by MNX1-AS1 knockdown.** (**A**-**C**) A549 control cells and MNX1-AS1-knockdown cells transfected with vector expressing ABCG2 or control vector were treated with paclitaxel (PTX) for 24 hours. The cell viability was detected by MTT assay (**A**). The apoptosis was detected by AO/EB double staining (**B**) and TUNEL assay (**C**). Scale bar: 50 μm. (**D**) Western blot detecting the expression of apoptosis-related proteins in A549 control cells and MNX1-AS1-knockdown cells transfected with vector expressing ABCG2 or control vector. β-actin or GAPDH was used as the loading control. Data were presented as mean ± S.D. *P < 0.05, **P < 0.01.

Finally, we aimed to evaluate whether ABCG2 is required for MNX1-AS1-mediated apoptosis in NSCLC cells. We transfected vectors expressing ABCG2 to MNX1-AS1-knockdown A549 cells and treated them with paclitaxel for 24 hours. MTT assay showed that re-expression of ABCG2 restored the viability of MNX1-AS1-knockdown A549 cells (Fig. 6A). Consistently, AO/EB double staining and TUNEL assays revealed that re-expression of ABCG2 resulted in decreased apoptosis of MNX1-AS1-knockdown A549 cells (Fig. 6B and C). Furthermore, we also examined the expression of apoptosis-related proteins and found that the up-regulated expression of BAX as well as cleaved caspase-3 and PARP1, caused by MNX1-AS1 knockdown, was reduced after re-expression of ABCG2 (Fig. 6D). Therefore, the above results indicate that ABCG2 is involved in MNX1-AS1 knockdown-caused apoptosis in NSCLC cells.

## Discussion

Lung cancer is the leading cause of cancer-related deaths worldwide and has a low 5-year survival rate, of which NSCLC is the predominant histological subtype 1,2,30. LncRNA has been reported to play essential roles in lung cancer development and progression 31-33. LncRNA MNX1-AS1 functions as an oncogene in various human cancers, including lung cancer 15-23, but its role in apoptosis, especially in ferroptosis, remains unclear. In this study, we found that knockdown of MNX1-AS1 could intensify RSL3-induced ferroptosis and paclitaxel-induced apoptosis by regulating the expression of ACSL4 and ABCG2.

Ferroptosis is an iron-dependent from of RCD driven by excessive lipid peroxidation on cellular membranes, which is mechanistically and morphologically different from apoptosis and other forms of RCD 5. Ferroptosis shows great potential in cancer therapy, especially in re-sensitizing therapy-resistant cancer cells to death 34,35. To investigate the role of MNX1-AS1 in ferroptosis in NSCLC cells, we here treated NSCLC control cells and MNX1-AS1-knockdown cells with RSL3, an important ferroptosis inducer, and found that the viability of MNX1-AS1-knockdown NSCLC cells was significantly decreased in a dose-dependent manner. Moreover, the levels of MDA and ROS in MNX1-AS1-knockdown NSCLC cells were higher than those in control cells upon RSL3 induction. These data suggest that MNX1-AS1 can regulate NSCLC ferroptosis and knockdown of MNX1-AS1 increases the sensitivity of NSCLC cells to ferroptosis.

We also studied the role of MNX1-AS1 in paclitaxel-induced apoptosis in NSCLC cells. Paclitaxel is usually used as the first-line anti-tumor drug in lung cancer, inhibiting proliferation of cancer cells by inducing cell cycle arrest and programmed cell death, particularly apoptosis 36, but its resistance is a great obstacle to the successful treatment 37. By adding paclitaxel to NSCLC control cells and MNX1-AS1-knockdown cells, we found that the viability of MNX1-AS1-knockdown NSCLC cells was lower than that in control cells. Furthermore, AO/EB double staining, TUNEL assay and Annexin V/PI double staining showed that knockdown of MNX1-AS1 significantly promoted the paclitaxel-induced apoptosis. Collectively, these data indicate that MNX1-AS1 plays an important role in both ferroptosis and apoptosis. LncRNAs, such as LINC00618 and P53RRA, have been reported to promote ferroptosis and apoptosis in cancer cells 38,39. Importantly, when ferroptosis inducer was used in combination with apoptosis inducer, the viability of MNX1-AS1-knockdown cells was further reduced, indicating that targeting MNX1-AS1 could further increase the sensitivity of NSCLC cells to the combination of ferroptosis inducer and apoptosis inducer. It has been reported that targeting ferroptosis is an effective strategy to overcome cancer resistance to paclitaxel 40,41. Furthermore, low-concentration paclitaxel and RSL3 shows synergistically inhibitive effect on tumor cell growth by inducing ferroptosis 42. However, whether MNX1-AS1 is involved in paclitaxel-induced ferroptosis is unclear now.

The regulatory roles of MNX1-AS1 in ferroptosis and apoptosis may be mediated by different signaling pathways. Our transcriptome sequencing data showed a large amount of DEGs when MNX1-AS1 was knocked down. Among these DEGs, ACSL4 and ABCG2 were validated to be involved in MNX1-AS1-mediated ferroptosis and apoptosis, respectively. ACSL4 is a lipid metabolism enzyme, which promotes ferroptosis by generation of lipid peroxidation 26-28. ABCG2, located mainly on the cell membrane, is a member of the ABC transporter superfamily, which effluxes a variety of substances, such as many common therapeutic drugs, and thus participates in drug resistance 29,43-45. Of course, our current results cannot rule out that there is a crosstalk between the two target genes. Whether ACSL4 is also involved in MNX1-AS1-mediated apoptosis, and whether ABCG2 is in turn involved in MNX1-AS1-mediated ferroptosis is not clear now, and it is worth exploring in the future. It has been reported that higher ACSL4 expression is associated with better survival in breast cancer patients receiving paclitaxel-cisplatin-based neoadjuvant chemotherapy 46.

Taken together, our findings showed that knockdown of MNX1-AS1 induces ferroptosis and apoptosis by regulating ACSL4 and ABCG2. This study highlights the role of MNX1-AS1 in ferroptosis and apoptosis, and provides MNX1-AS1 as a potential target for tumor therapy.

### Limitations of the study

In this study, ACSL4 and ABCG2 were identified as downstream effectors of MNX1-AS1; however, the underlying mechanism by which MNX1-AS1 regulates ACSL4 and ABCG2 expression is unclear.

## Supplementary Material

Supplementary table.

## Abbreviations

NSCLC: Non-small cell lung cancer
LncRNA: Long non-coding RNA
RCD: Regulated cell death
MDA: Malondialdehyde
ATCC: American Type Culture Collection
ROS: Reactive oxygen species
AO/EB: Acridine orange/ethidium bromide
TUNEL: Terminal deoxynucleotidyl transferase-mediated dUTP nick-end labeling
PTX: paclitaxel
RT-qPCR: Reverse transcription-quantitative real-time polymerase chain reaction
