# Supplementary material for: Downregulation of lncRNA MNX1-AS1 promotes the ferroptosis and apoptosis of non-small cell lung cancer
Source: Int J Med Sci. 2025 Feb 3;22(5):1052–63. doi: 10.7150/ijms.97790 (PMC11866536; doi:10.7150/ijms.97790)
Supplement: Supplementary file 1 — Supplementary table. [file ijmsv22p1052s1.pdf]

Primer name	Forward primer sequences (5′-3′)	Reverse primer sequences (5′-3′)
MNX1-AS1	CGCATTTTCAGATTCACGCAGG	TTGCAAGACTCACGTAGCACT
GAPDH	GCTGAGAACGGGAAGCTTGT	GCCAGGGGTGCTAAGCAGTT
ACSL4	GACTGGCAAGAAGGCGGTTATAC	GGAGATGTTCTGTCCACCAATTACG
EMP1	GTCATTATGCGAATCGTGATGGAAC	CAGATAGAGAACGCCGATGATGAAG
MEG3	CAGACGGCGGAGAGCAGAG	AAGACAAGGAGGTGGACGGATG
TRIM36	ATGAACACCTCCTGCTGAACTTG	TGCCAGTATCTCCAATGATGTAGTC
GEM	CACCAGTACAGCCACCGCAAC	TTCTCCCAGCACCTCGCAGTC
STAT3	ACCAAGCGAGGACTGAGCATC	CAGCCAGACCCAGAAGGAGAAG
SQLE	ACCCGAGTCCAGTTCTCATCTACC	AAGCAACAGTCATTCCTCCACCAG
NRP2	TTGATGATGAATACGAGGTGGACTG	GCGATGATGGTGATGAGGATGG
BDNF	AGAGGCTTGACATCATTGGCTGAC	CGCCGTTACCCACTCACTAATACTG
DYRK2	TTCACCTTCCGCAACCACATCTG	CCGCTTCTACCCTGCTGCTTTAAC
MXD1	AACAGCAGTAGCAGATCAACTCAC	CTCCAGGCACAAGCGAAGATG
STRA6	CCTTGGTCTTGTCCTGCTTACTCAC	AGGCTGTCTGGTAGGCACTGAAG
PAM	TGTTGGATTCAGAGTTGGAGGAGAG	TGGCAGACGTGTGAGGTGTAAG
FOXC1	CGCCACAACCTCTCGCTCAAC	TGTCCTTCTCCTCCTTGTCCTTCAC
DAPK1	CTGCTGGTGCTGCTGGTCAAC	GCTCAGGTAATGCTTCACGGTCAG
CDC7	GCCTGACCTGTGACTGCTATGC	CTGGTGCTCTGAATCCTGGTGT
CYP24A1	TCGCTTGCCGCCTTCCTG	ACGCCGTAGATGTCACCAGTC

ASS1	GGCTGAAGGAACAAGGCTATGAC	CCACAAACTCCCTGCTGACATC
ABCG2	GGTCAGAGTGTGGTTTCTGTAGCA	GTGAGAGATCGATGCCCTGCTT
